# Nanosized copper particles induced mesangial cell toxicity via the autophagy pathway

**DOI:** 10.1590/1414-431X2022e12252

**Published:** 2022-11-11

**Authors:** Pengjuan Xu, Min Cao, Xueqian Dong, Zhichao Yu, Jianwei Liu, Junzhen Tan, Yiyi Wang, Tao Li, Shuwu Zhao

**Affiliations:** 1 School of Integrative Medicine, Tianjin University of Traditional Chinese Medicine, Tianjin, China; 2 Scientific Research Office, Tianjin Sino-German University of Applied Sciences, Tianjin, China; 3 Department of Neurology, Tianjin Haihe Hospital, Tianjin, China

**Keywords:** Nanosized copper particles (nano Cu), Mesangial cells (MCs), Autophagy pathway, Autophagic flux, Nephrotoxicity

## Abstract

Nanosized copper particles (nano Cu) have been incorporated into products in multiple industries, although studies have demonstrated that these particles are nephrotoxic. We investigated the cytotoxicity of nanosized copper particles on rat mesangial cells and measured rates of apoptosis, the expression of caspase-3, and generation of reactive oxygen species. We also measured autophagy through the acridine orange (AO) staining and expression of Beclin-1, microtubule-associated protein 1 light chain 3, and p62 to screen the underlying mechanism of toxicity. Nanosized copper particles inhibited mesangial cell viability, up-regulated the activity of caspase-3, and increased the rates of apoptosis and the generation of reactive oxygen species in a concentration-dependent manner. Exposure to nano Cu increased the formation of acidic vesicular organelles and the expression of Beclin-1, microtubule-associated protein 1 light chain 3, and p62, and treatment with an autophagy inhibitor reduced nephrotoxicity. This indicated that the autophagy pathway is involved in the toxicity induced by nanosized copper particles to mesangial cells. This finding can contribute to the development of safety guidelines for the evaluation of nanomaterials in the future.

## Introduction

Nanosized materials have structural components smaller than 100 nm in at least one dimension. Because of their unique size and high surface area, nanosized materials are widely used in various chemical, physical, and biological fields. With the development of nanotechnology, more and more nanosized materials have been introduced into our daily life. Nanosized materials can cross the blood-brain barrier and the blood-testis barrier after surface modifications (1,2). Therefore, the urgency to ascertain the safety of nanomaterials has been recognized by the scientific community and governments. The United States Environmental Protection Agency has reported that artificial nanoparticles may be toxic (3).

Nanosized copper particles (nano Cu) are metallic nanomaterials that are used in the textile industry, energy production, and agriculture (4-6). Their antimicrobial properties have leveraged their application in biomedicine, medical devices, and wound dressings, which may lead to an increased risk of exposure (7,8). Despite the advantages of nano Cu, the understanding of toxicology and cellular mechanism is extremely limited. Nano Cu can enter the body by inhalation, dermal penetration, ocular exposure, and ingestion (9). Following uptake, nano Cu can translocate into organs such as the brain, liver, and lungs, which can potentially induce pathological changes and organ injuries. Nano Cu has been reported to damage the spleen and immune system (10), kidneys, and liver in rats following oral administration (11). Ingestion of nano Cu severely affects hepatic drug metabolism in rats by inhibiting the expression of cytochrome P450 enzymes (12). Normal renal function involves the filtration of waste products from the blood. For this reason, the kidney is the organ most affected by nanosized materials, and also the target organ for accumulation (13). As resident cells of the kidney, glomerular mesangial cells (MCs) are the main cellular constituents in the glomerulus, which account for approximately 30-40% of the total cells in the glomerulus. Together with the mesangial matrix, MCs form the glomerular basement membrane (GBM) in the glomerulus, and they also play a crucial role in maintaining glomerular function (14). Much evidence indicated that MCs participate in the pathological changes of various kidney diseases.

When a drug or harmful substances accumulate in the kidney, MCs are often affected, which can lead to functional transformation (15).

Autophagy is a lysosome-based degradative pathway involved in multiple cellular processes and linked to various diseases (16). Autophagy maintains homeostasis by eliminating excessive or unnecessary proteins and injured or aged organelles in normal cells. The rate of autophagosome formation is relevant to the pathogenesis of disease, and autophagy has become a focus of nanotoxicity research (17). Carbon-based nanosized materials, silica nanosized materials, quantum dots, and rare earth oxide nanosized materials have all been shown to induce autophagy dysregulation and cell death (18,19).

To date, the interaction of nano Cu with mesangial cells and its effects on autophagy have not been investigated. We used the HBYZ-1 rat mesangial cell line to assess the effects of nano Cu on cytotoxicity and autophagy in these cells to provide new insights into the nephrotoxicity of nano Cu and suggest a possible pathway for relieving the injury induced by nano Cu in the kidney.

## Material and Methods

### Materials

Nano Cu particles were obtained from Macklin Biochemical Co., Ltd., China (CAS:7440-50-8) at 99.9% purity, and Hoechst 33342, acridine orange (AO), and 3-methyladenine (3-MA) were obtained from Sigma Chemical Co. (USA). DMEM medium and fetal bovine serum (FBS) were obtained from Gibco BRL (USA). Cell Counting Kit-8 (CCK-8) assay kit was obtained from Dojindo Molecular Technologies, Inc. (China). The Annexin V-FITC propidium iodide (PI) apoptosis detection kit was obtained from Beyotime Biotechnology (China). The enzyme-linked immunosorbent assay (ELISA) kit for caspase-3 was purchased from Cloud-Clone Corp., formerly Uscn Life Science Inc. (China). The ROS testing kit was from Genmed Scientifics Inc. (USA). Rabbit monoclonal anti-Beclin-1, rabbit polyclonal anti-LC 3B antibody, rabbit polyclonal to Akt, rabbit monoclonal anti-p70S6K, and rabbit monoclonal anti-mTOR were purchased from Abcam (USA). The rabbit monoclonal anti-p62/SQSTM1 antibody was obtained from Abways Technology, Inc. (China). The rabbit anti-phosphorylated-p70S6K (anti-p-p70S6K), rabbit monolyclonal anti-phosphorylated-Akt (anti-p-Akt), and rabbit monolyclonal anti-phosphorylated-mTOR (anti-p-mTOR) antibodies were obtained from Cell Signaling Technology, Inc. (USA). Mouse monoclonal anti-GAPDH antibody was obtained from OriGene Technologies, Inc. (USA), and the chemiluminescent HRP substrate (Immobilon western) was purchased from Millipore Corporation (USA).

### Cell culture

The rat mesangial cell line (HBYZ-1) was obtained from China Center for Type Culture Collection (China). Cells were cultured in DMEM in an incubator aerated with 95% humidified air/5% CO_2_ at 37°C, supplemented with 10% FBS and 1% antibiotics (penicillin and streptomycin, Cyagen Biosciences (Guangzhou) Inc., China).

### Pretreatment of nano Cu

Before experiments, nano Cu was floated using the culture media and dispersed for 20 min by a sonicator to prevent aggregation, and it was diluted to different concentrations with DMEM medium.

### Characterization of nano Cu

In order to measure particle size, nano Cu was examined under a transmission electron microscope (TEM) (Tecnai G2 F30 S-TWIN, FEI, USA) and through scanning electron microscope analysis (SEM) (SU8020, Hitachi, Japan). The distribution of nano Cu average size in culture medium and polymer dispersion index (PdI) were determined by dynamic light scattering (DLS).

### Cell viability assay

The cytotoxicity induced by nano Cu on MCs was assessed by the Cell Counting Kit-8 assay, which is widely used to evaluate cytotoxic activity.

Cells were exposed to concentrations of 0, 1, 10, 30, and 50 μg/mL nano Cu for 1, 2, 3, 6, and 12 h, according to our previous work (20). Subsequently, CCK-8 reagent was added to each well, and the 96-well plate was incubated for 1.5 h at 37°C. Absorbance was measured at a wavelength of 490 nm. Cell viability is reported as a percentage of the control culture viability. Each condition and experiment were performed in triplicate.

### Detection of apoptotic cells

The apoptotic index of nano Cu was assessed using Annexin V-FITC and PI staining and analyzed by flow cytometry. After different concentrations of nano Cu or cell culture medium treatment for 3 h, cells were harvested and incubated with PI and V-FITC, according to the manufacturer's instructions. The apoptotic rate was determined by flow cytometry and calculated as follows: apoptosis ratio (%) = (percentage of early apoptotic cells) + (percentage of late apoptotic cells). All experiments were performed in triplicate.

### Hoechst 33342 staining

The changes of cells nuclei under nano Cu treatment were detected using Hoechst 33342 staining. Cells were exposed to various concentrations of nano Cu for 3 h. Then, after incubating with Hoechst 33342 dye solution for 20 min in the dark, the sample was observed under a laser confocal fluorescence microscope (Leica, Germany). All experiments were performed in triplicate. The percentage of apoptotic cells was calculated as followed: apoptotic cells (%) = (number of apoptotic nuclei) / (number of total cell nuclei) × 100.

### ELISA assay for caspase-3 activity

The activity of caspase-3 in MCs was measured by the enzyme-linked immunosorbent assay employing a commercial ELISA kit. MCs were plated onto 6-well plates and treated with different concentrations of nano Cu for 3 h. The cells underwent trypsin digestion and were lysed with lysate, and then centrifuged at 13,800 *g* for 10 min at 4°C to harvest the liquid supernatant. Afterward, the activity of caspase-3 was measured in the supernatant according to the manufacturer's instructions.

### Measurement of ROS generation

The intracellular reactive oxygen species (ROS) was measured by dichlorofluorescein diacetate (DCFH-DA). After the MCs were exposed to different concentrations of nano Cu (0, 1, 10, 30, and 50 µg/mL) for 3 h, cells were washed and incubated in DCFH-DA working solution for 30 min. DCFH-DA entered the cells and combined with the intracellular reactive oxidoreductase to form the fluorescent compound dichlorofluorescein diacetate. Flow cytometry was used to detect the intracellular accumulation of ROS, and the level of ROS as the percentage of DCF-positive cells in M1 region. Each condition and experiment were performed in triplicate.

### Acridine orange (AO) staining

The effect of nano Cu on the acidic vesicle organelles was assessed using AO staining, which was used to verify the autophagy markers in cells. Following treatment with nano Cu (0, 1, 10, 30, and 50 µg/mL) for 3 h, cells were stained with 1 µM AO for 20 min at 37°C. Then, cells were observed using a fluorescence microscope.

### Western blotting assay

MCs were incubated with different concentrations of nano Cu (0, 10, 30, and 50 µg/mL) for 3 h. Sample proteins were extracted and protein concentrations were measured using the BCA assay kit (Beyotime Biotechnology). Then, the proteins were used for SDS-polyacrylamide gel electrophoresis (SDS-PAGE), and the separated proteins were transferred to a polyvinylidene difluoride (PVDF) membrane, blocked in 5% non-fat milk for 2 h at room temperature, and then incubated with primary antibody (anti-Beclin-1, LC3, p62, mTOR, p-mTOR, Akt, p-Akt, p70S6K, or p-p70S6K) overnight at 4°C. The membranes were incubated with secondary antibodies for 1 h at room temperature. The target protein was detected using a chemiluminescent HRP substrate detection kit. The bands were analyzed by ImageJ software (NIH, USA), and normalized to GAPDH. All experiments were performed in triplicate.

### Effect of 3-MA

In order to assess the effect of autophagy on the toxicity of nano Cu in MCs, 3-MA (an autophagy inhibitor, 10 μmol/L) was used. MCs were exposed to 3-MA for 1 h before the treatment of 30 µg/mL nano Cu according to reference 21[Bibr B21]. Cell viability was determined by CCK-8.

Each condition and experiment were repeated three times.

### Statistical analysis

All data are reported as means±SD and were analyzed by Origin 9.0 (www.originlab.com) and SPSS 22.0 (IBM, USA). Statistical analysis was performed using one-way ANOVA and P<0.05 was considered as significant.

## Results

### Characterization of nano Cu

**Figure 1:**
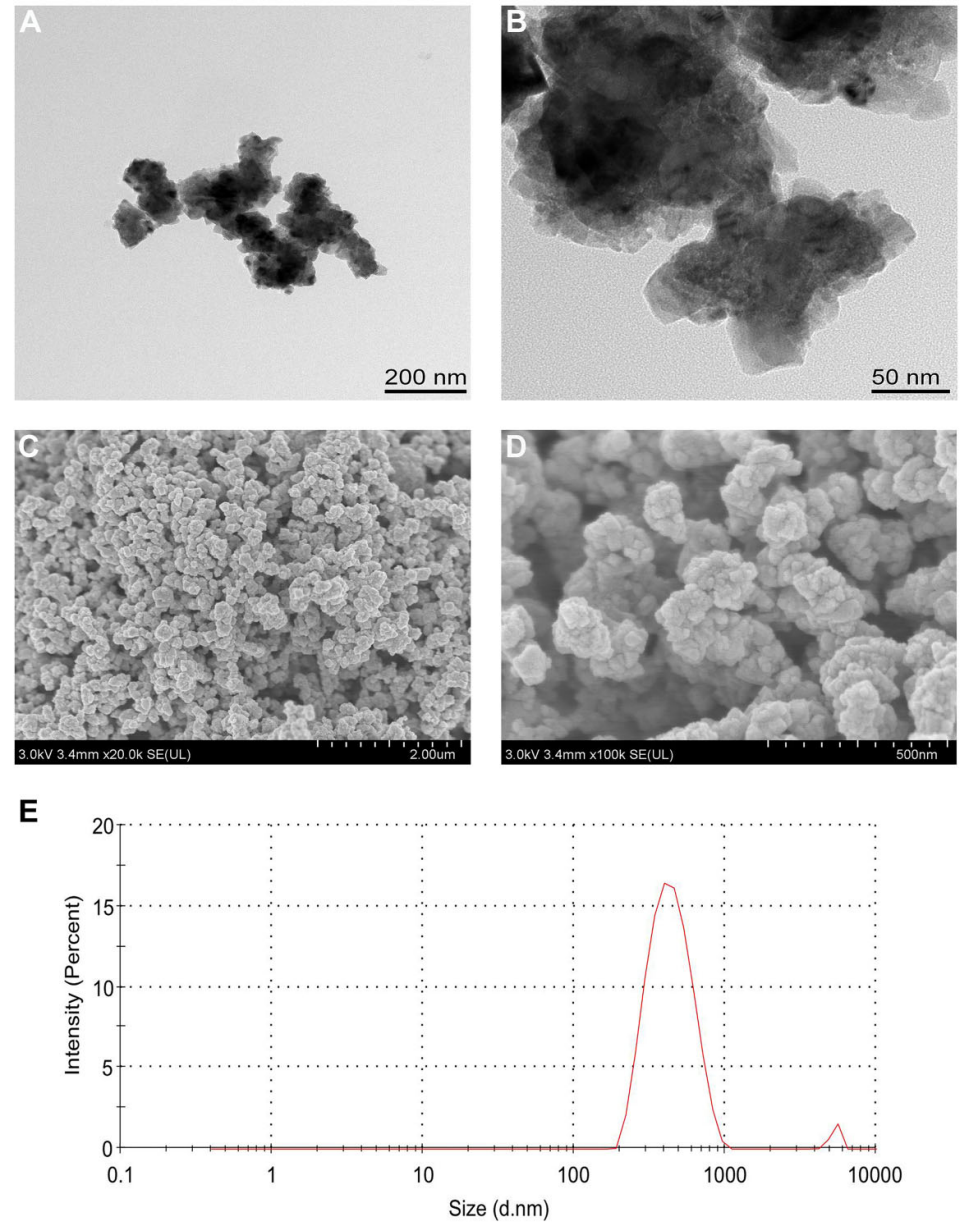
Characterization of nanosized copper particles (nano Cu). **A** and **B**, Transmission electron microscope images of nano Cu. **C** and **D**, Scanning electron microscopy images of nano Cu. Scale bars: **A**, 200 nm; **B**, 50 nm; **C**, 2.00 μm; **D**, 500 nm. **E,** Distribution of nano Cu average size in culture medium was determined by dynamic light scattering image.

In microscopy images, nano Cu particles appeared as a red-brown to black powder, as shown in Figure 1. TEM images revealed that the average diameter of nano Cu was in the range of 30-60 nm. Then, SEM was used to observe the surface morphology of nano Cu. Figure 1C and D show that the nanoparticles possessed an irregular spherical shape and were well dispersed. They also had a rough surface. In addition, particle size had an average of 449 nm, and PdI was 0.238.

### Nano Cu reduced cell viability of MCs

**Figure 2:**
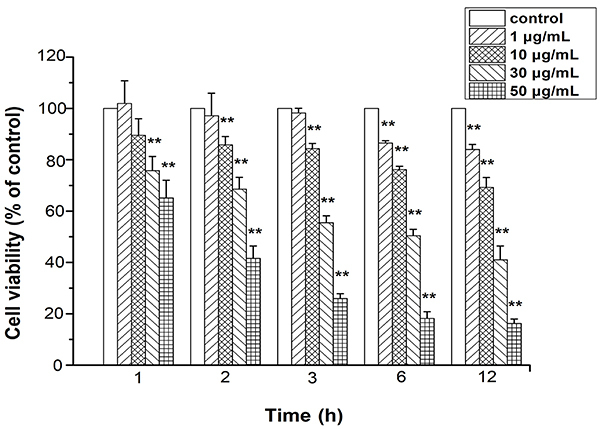
Effect of nanosized copper particles (nano Cu) on cell viability in mesangial cells (MCs). A culture medium (0, 1, 10, 30, and 50 μg/mL nano Cu) was used to treat the MCs for 1, 2, 3, 6, and 12 h. A CCK-8 assay was used to measure the viability of the cells, and the outcomes are reported as the absorbance percentage relative to the control group. Data are reported as means±SD (n=6/group). **P<0.01 *vs* control group (ANOVA).

Nano Cu treatment significantly decreased cell viability compared with control (Figure 2). Cell death induction by nano Cu was concentration- and time-dependent. The incubation time of 3 h was selected for the different concentrations of nano Cu in the subsequent experiments according to the preliminary experiment and our previous work (20).

### Nano Cu induced apoptosis of MCs

**Figure 3:**
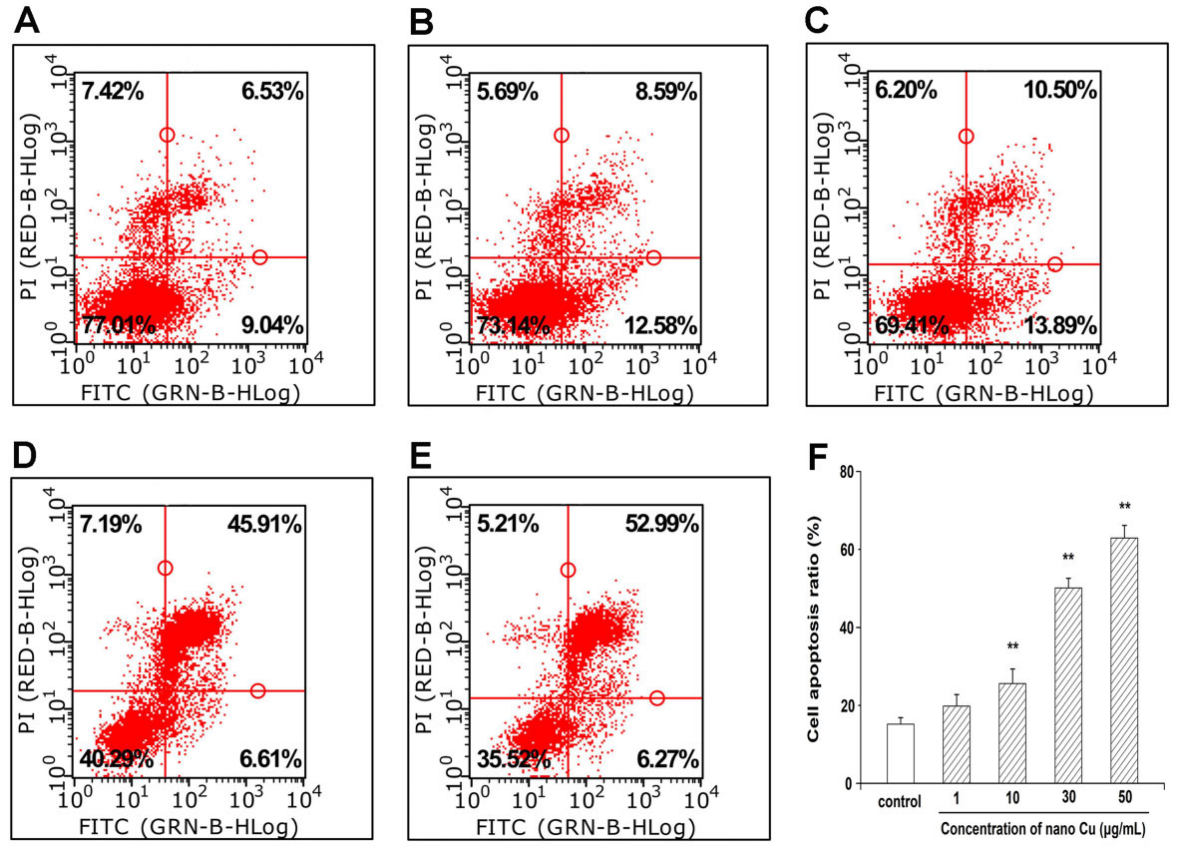
Effect of nanosized copper particles (nano Cu) on cell apoptosis in mesangial cells (MCs) by flow cytometry analysis. Cells were exposed to 0, 1, 10, 30, and 50 μg/mL nano Cu for 3 h. **A**, Control group. **B**-**E**, Cells treated with 1, 10, 30, and 50 µg/mL nano Cu, respectively. **F**, Bar graph of the results. Data are reported as means±SD, from three independent experiments. **P<0.01 *vs* control group (ANOVA).

Apoptosis was investigated by flow cytometry and Hoechst 33342 staining. As shown in Figure 3, the exposure of MCs to nano Cu increased the percentage of cell apoptosis, and this increased in a dose-dependent manner.

**Figure 4:**
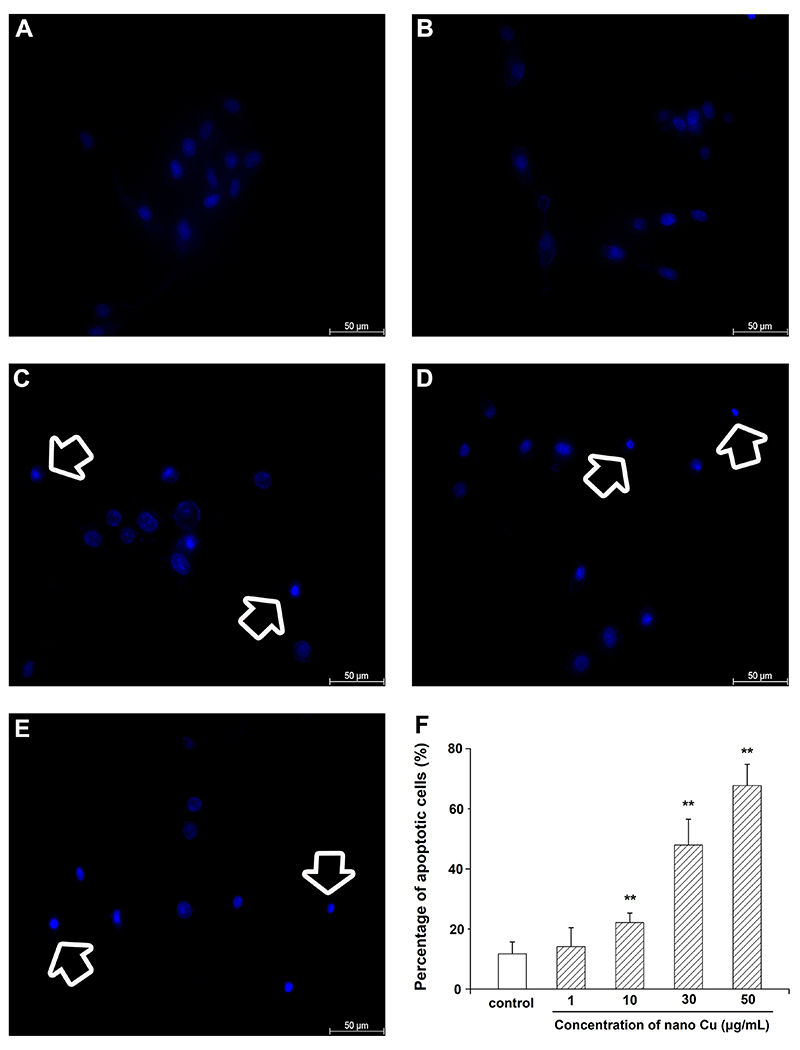
Effect of nanosized copper particles (nano Cu) on cell morphological characteristics of apoptosis by Hoechst 33342 staining in mesangial cells (MCs). Cells treated using nano Cu for 3 h were stained with Hoechst 33342 and images were observed under the fluorescence microscope. **A**, Control group. **B**-**E**, Cells treated with 1, 10, 30, and 50 µg/mL nano Cu, respectively. The white arrows show nuclear condensation and fragmentation in MCs. Scale bar, 50 µm. **F**, Bar graph of the results. Data are reported as means±SD, from three independent experiments. **P<0.01 *vs* control group (ANOVA).

As a nuclear fluorochrome, Hoechst 33342 staining was used to diagnose the morphological characteristics of apoptosis at different nano Cu concentrations. The results showed that nano Cu significantly induced chromatic agglutination and apoptotic body formation, in a dose-dependent manner (Figure 4).

### Nano Cu increased the activity of caspase-3 in MCs

**Figure 5:**
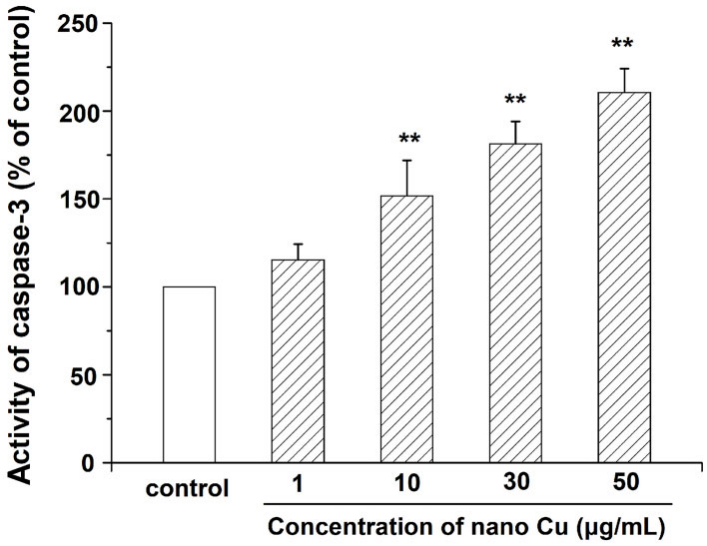
Effect of nanosized copper particles (nano Cu) on the activity of caspase-3 in mesangial cells (MCs) by ELISA. MCs were treated with different concentrations of nano Cu (0, 1, 10, 30, and 50 μg/mL) for 3 h. Data are reported as means±SD, from three independent experiments. **P<0.01 *vs* control group (ANOVA).

Accumulated evidence has demonstrated that caspase-3 induces cell apoptosis and activated caspase-3 is responsible for the cell entering the irreversible stage of apoptosis. As shown in Figure 5, the level of caspase-3 in nano Cu-conditioned MCs was higher than that in untreated MCs. These results indicated that nano Cu could induce toxicity in MCs by decreasing the viability and increasing the apoptosis.

### Nano Cu increased the generation of ROS in MCs

**Figure 6:**
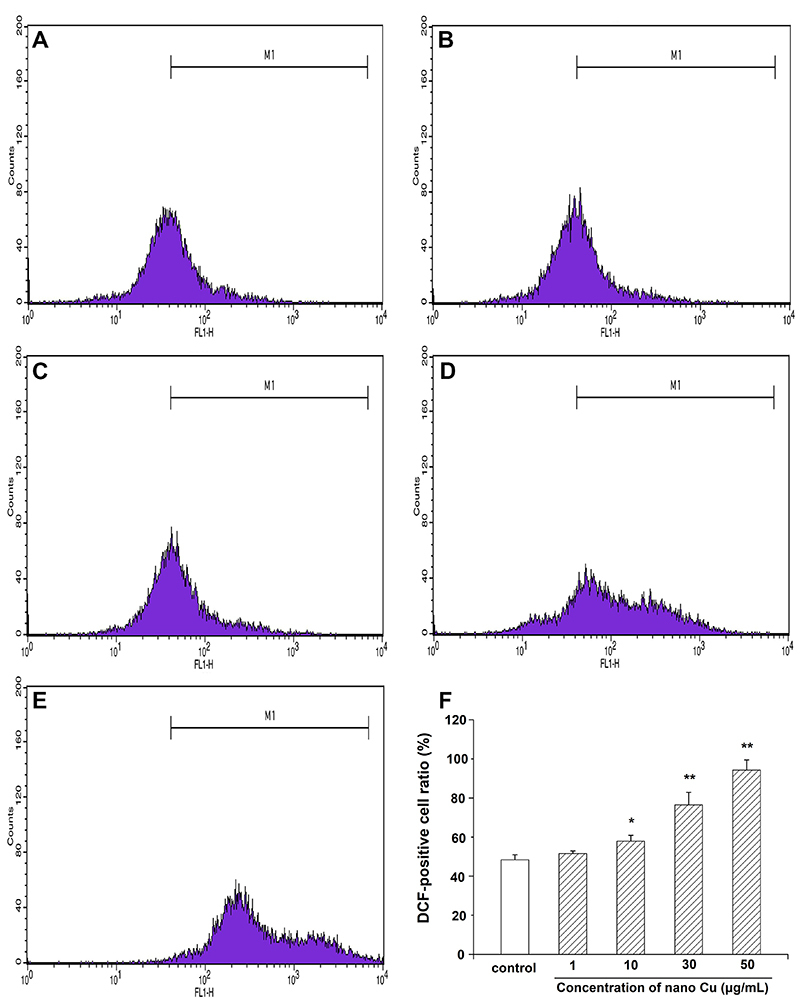
Effect of nanosized copper particles (nano Cu) on the generation of reactive oxygen species in mesangial cells (MCs) by DCFH-DA staining with flow cytometry analysis. Cells were exposed to 0, 1, 10, 30, and 50 μg/mL nano Cu for 3 h and fluorescence intensity was measured. **A**, Control group. **B**-**E**, Cells treated with 1, 10, 30, and 50 µg/mL nano Cu, respectively. **F**, Bar graph of the results. Data are reported as means±SD, from three independent experiments. *P<0.05, **P<0.01 *vs* control group (ANOVA).

As shown in Figure 6, the generation of ROS was significantly increased with increasing doses of nano Cu compared with the control group. Collectively, the result suggested that nano Cu induced injury in MCs in a dose-dependent manner.

### Nano Cu blocked autophagic flux in MCs

**Figure 7:**
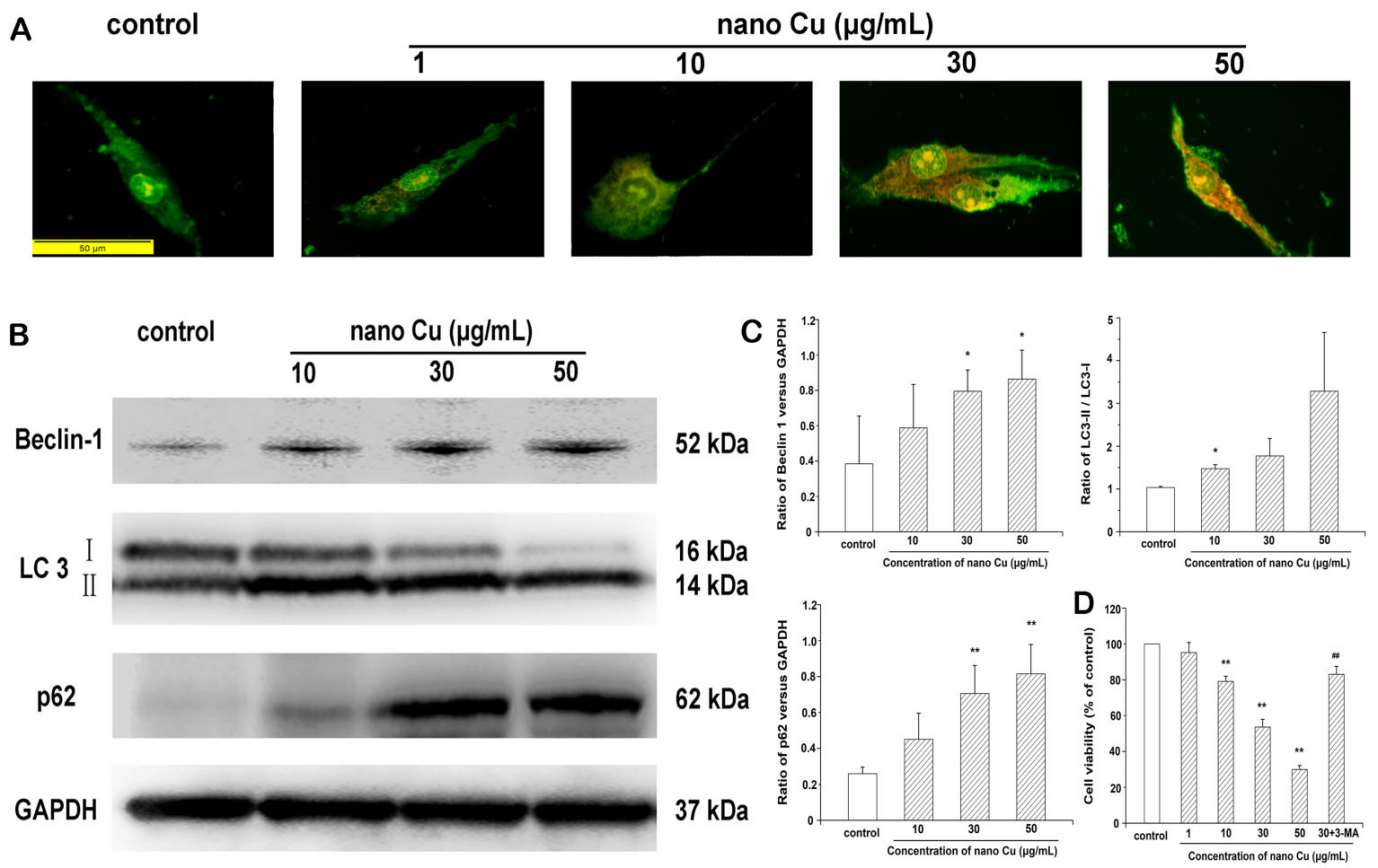
Effect of nanosized copper particles (nano Cu) on autophagy in mesangial cells (MCs). **A**, Effect of nano Cu on the formation of acidic vesicular organelles in MCs. Cells were treated with nano Cu for 3 h, stained by acridine orange. The formation of acidic vesicular organelles was examined under fluorescence microscopy. Scale bar is 50 µm. **B**, Cells were subjected to Western blotting analysis with anti-Beclin-1, anti-LC3, and anti-p62 antibodies, after exposing to 0, 10, 30, and 50 μg/mL nano Cu for 3 h. GAPDH served as the loading control. **C**, The corresponding linear diagram of immunoblotting quantitation was shown. Each reported value represents as means±SD. From three independent experiments. **D**, Effect of 3-MA on the cytoxicity of nano Cu in MCs. A CCK-8 assay was used to detect the effect of 3-MA on the cytoxicity of nano Cu on cell viability in MCs. Cells were exposed to cell culture medium, 1, 10, 30, and 50 μg/mL nano Cu, and 30 μg/mL nano Cu+3-MA for 3 h. Data are reported as means±SD (n=6/group). *P<0.05, **P<0.01 *vs* control group. ^##^P<0.01 *vs* 30μg/mL nano Cu group.

As shown in Figure 7A, the control cells displayed green fluorescence with minimal red fluorescence, and nano Cu-treated MCs showed an increase in red fluorescence, which meant that nano Cu induced the accumulation of acidic vesicular organelles. The expression of Beclin-1 and LC3 II/I ratio in the MCs treated with nano Cu was significantly upregulated compared with control. However, the p62 level was significantly increased in the nano Cu groups. These results therefore demonstrated that nano Cu treatment caused autophagosome accumulation and impaired the autophagic degradation capacity.

To confirm that increased autophagic activity is associated with cell death, we used the CCK-8 assay to assess the role of autophagy in nano Cu cytotoxicity. MCs were exposed to increasing concentrations of nano Cu and 3-MA. Pretreatment with 3-MA significantly increased cell viability compared with that of 30 µg/mL group. These results suggested that autophagy played a pro-death role in nano Cu-induced MCs death.

### Nano Cu inhibited the Akt/mTOR/p70S6K pathway in MCs

**Figure 8:**
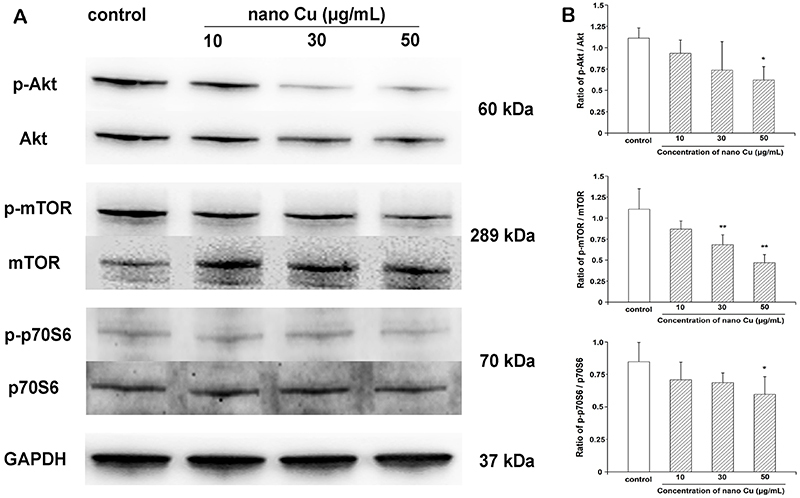
Effect of nanosized copper particles (nano Cu) on the Akt/mTORp70S6K pathway in mesangial cells (MCs). **A**, MCs were exposed to 0, 10, 30, and 50 μg/mL nano Cu for 3 h and subjected to western blotting analysis with anti-Akt, anti-p-Akt, anti-mTOR, anti-p-mTOR, anti-p70S6K, and anti-p-p70S6K antibodies. GAPDH served as the loading control. **B**, Corresponding linear diagrams of immunoblotting quantitation. Data are reported as means±SD, from three independent experiments. *P<0.05, **P<0.01 *vs* control group (ANOVA).

As shown in Figure 8, the level of phosphorylated Akt was significantly decreased by treatment with nano Cu. Similarly, the expressions of p-mTOR and p-p70S6K were significant reduced in the nano Cu group, compared with control. There were no changes in the levels of total Akt, mTOR, and p70S6K. These results confirmed that nano Cu could inhibit the Akt/mTOR/p70S6K pathway in MCs.

## Discussion

The production and application of nanosized materials in various fields has been rapidly increasing in recent years, but little is known about their effects at the molecular level. Nano Cu is a potential anti-bacterial, anti-viral, antibiotic, and growth-promoting substance with broad applications (7,22). However, its toxicity has been demonstrated in plants and animals, which precludes large-scale application (10,23). The kidney is one of the target organs of nano Cu and its cytotoxic effects have caused serious injuries to cells in kidney (20,24). We found that nano Cu is markedly cytotoxic to mesangial cells in a concentration- and time-dependent manner. Previously, many reports have shown induction of ROS-mediated cell death in various cells by different nanoparticles. ROS is closely associated with oxidative stress, and its level is generally considered to reflect cell injury. In this study, nano Cu increased apoptosis and induced oxidative damage, as shown by the levels of ROS, correlating with our previous findings (20).

Several studies have demonstrated that apoptosis and autophagy can promote or inhibit each other (25). Further experimental research is needed to study the molecular mechanism between apoptosis and autophagy during the effect of nanoparticles. Autophagy has been shown to be involved in the toxicity of nanoparticles, and ROS are known inducers of autophagy (26,27). Zinc oxide nanoparticles and single-walled carbon nanotubes (SWCNTs) have been reported to cause autophagy in rat tracheal epithelial cells (28), and silver nanoparticles can induce cytotoxicity in mouse hippocampal neuronal HT22 cells by increasing autophagy (29). We were interested in knowing whether autophagy is involved in the nano Cu-induced apoptosis of MCs.

AO is a cell-permeable green fluorophore, which can shift to red fluorescence in the acidic environment. Therefore, AO staining is a reliable method to assess the level of autophagy. Our results showed that nano Cu induced the accumulation of acidic vesicular organelles. Based on this, we concluded that nano Cu upregulated the level of autophagy. Beclin 1 is critical to autophagy and LC3 is essential for the dynamic process of autophagosome formation. LC3 can covalently conjugate to phosphatidylethanolamine (PE), and modify to a membrane-bound form (LC3-II) (30). LC3-II is localized in pre-autophagosome and autophagosome membranes and is widely used as an autophagosome marker in mammalian cells (31). We found a marked increase in the expression of Beclin 1 and conversion of LC3-II, which was also concentration-dependent, suggesting that autophagosome accumulation is the result of exposure to nano Cu.

Cell stress induces autophagy and increases the rate of autophagosome formation and accumulation. However, the accumulation of autophagosomes can be caused by the induction of autophagy or the blockage of autophagosomal maturation and degradation (31). Therefore, we further analyzed the autophagic degradation of p62, also known as sequestome 1 (SQSTM1). p62 is a scaffold protein that can bind directly to LC3 and recruit LC3 to the autophagic machinery for degradation. Studies have pointed out that the expression level of p62 inversely correlates with autophagosomal maturation and degradation (32,33). Exposure to nano Cu resulted in significant accumulation of p62, indicating an inhibition of autophagic degradation.

To further confirm our conclusion that nano Cu treatment can induce autophagy and block the autophagic flux, we pre-treated mesangial cells with an autophagy inhibitor, which reduced cell viability. Altogether, the data suggested that nano Cu caused autophagosome accumulation by activating autophagy and blocking autophagic flux.

It is well known that the major function of autophagy is to fight against various forms of injury to keep cells alive (34). Apoptosis, known as type I programmed cell death, is the sacrifice of specific cells for the greater benefit of the organism (35). However, in the context of nanotoxicity, autophagy has shown to play a puzzling role. Nanoparticle-induced autophagy induction has a pro-cell death role in nanotoxicity (17,36). We also confirmed that nano Cu-induced dysregulation of autophagy promoted apoptosis and speculated that nano Cu-mediated autophagy might be an adaptive cellular response, which assisted in the clearance of nanoparticle during initial stages. But later it activated the excessive autophagy and blocked the autophagic flux, which might be harmful and cause cellular dysfunction. Furthermore, excessive autophagy or autophagy activity perturbation were arbiters in cell apoptosis and necrosis.

Thus far, as a classic signaling pathway, the mammalian target of rapamycin (mTOR) pathway plays a negative role in autophagy by regulating autophagy-related proteins and lysosome biosynthesis (37,38). Generally, mTOR phosphorylation can upregulate the phosphorylation of p70 ribosomal S6 kinase (p70S6K) under normal conditions and suppress autophagy. The phosphorylation of protein kinase B (Akt) regulates mTOR (39). In the current study, we analyzed the phosphorylation levels of Akt, mTOR, and p70S6K to assess the role of the Akt/mTOR/p70S6K pathway in nano Cu-induced autophagy and found that nano Cu reduced the level of p-mTOR, with similar findings for p-Akt and p-p70S6K. We therefore concluded that nano Cu induced autophagy through downregulation of Akt/mTOR/p70S6K pathway activation in mesangial cells. These experimental results provide a reference to the cytotoxicity of nano Cu, and we will next investigate whether activation of Akt/mTOR/p70S6K signaling can promote mesangial cell viability following exposure to nano Cu. Importantly, various upstream signaling pathways can inhibit or enhance autophagy levels by regulating mTOR (40). Thus, whether other pathways, such as oxidative stress, are involved in nano Cu-induced autophagy and cytotoxicity requires further study.

## Conclusion

In conclusion, we found that nano Cu induced apoptosis and decreased the viability of mesangial cells through autophagy and downregulation of p-AKT, p-mTOR, and p-p-70S6K proteins. The results showed that nano Cu treatment activated autophagy and blocked the autophagic flux through regulation of the Akt/mTOR/p70S6K pathway, which was previously not known.
